# ‘Just saying it like it is’: A comparative study on the characterizations of Chris Christie and Donald Trump as *tough-guy* politicians

**DOI:** 10.1177/09639470251404158

**Published:** 2025-11-29

**Authors:** Samuel Bourgeois

**Affiliations:** 1 School of English, 5289 Manchester Metropolitan University, Manchester, UK

**Keywords:** American political rhetoric, characterization, Chris Christie, Donald Trump, impoliteness

## Abstract

This article pursues two main research aims. First, it examines how two politicians running for president in 2016 strategically drew on stereotypical tropes of Metropolitan New Yorkers to characterize themselves as brash and argumentative ‘tough guys’ from New York City and North New Jersey respectively in an effort to project strength and effectiveness as leaders. Second, it tests the extent to which Donald Trump’s abrasive persona was truly unique and norm-breaking upon his entrance into politics. Through comparative case studies of Trump and New Jersey Governor Chris Christie – both known for characterizing themselves as New York/New Jersey tough guys during the 2016 Republican primaries – this study examines their rhetorical styles prior to and during the 2016 primary debates. While both figures attracted attention for their impoliteness and blunt speech, only Trump successfully translated this into political success in the primaries and general election alike. While Christie’s brash rhetorical use of impoliteness maintained an image closely tied to that of a New Jersey tough guy, Trump’s tough-guy persona became increasingly idiomatic as his career developed from the 2000s. Moreover, Trump is found to use impoliteness strategies that are more gratuitously face-aggravating and *ad hominem* in nature. The data therefore confirms that Trump indeed entered politics as a more brash, offensive and norm-challenging politician even when compared to others, including those from his home region.

## 1. Introduction

This paper conducts a comparative study of former Republican Governor of New Jersey and two-time presidential candidate Chris Christie and Donald Trump, the 45th and 47th President of the United States, of New York City (NYC).^1^ In addition to hailing from the New York Metropolitan area, both figures gained national attention before and during the 2016 Republican primaries for embodying what will henceforth be called the Metropolitan New Yorker *tough-guy persona* – a style characterized by several schematic elements commonly associated with quintessential New Yorkers and North New Jerseyans: being *brash*, *in-your-face*, *straight-talking* and unapologetically *telling it how it is*. Such characterizations of Christie fitting this description can be found in Christie (2019) and Siegel (2013), while D’Antonio (2016), O’Brien (2016 [2005]) and Wolff (2018) identify these schematic elements in Trump’s public persona. These reputations persist to this day for both figures.

This study has two main aims. First, it explores how NYC and North New Jersey politicians can use the local cultural schema of the straight-talking tough guy to project strength and leadership. Though often portrayed in fiction, this schema has only received limited scholarly attention, and its appropriation by public figures to entertain, connect with and gain support from their targeted audiences remains underexamined. This inquiry is timely given (a) the growing expectations on politicians to entertain the electorate (see Tracy, 2017: 742–743; Wodak et al., 2021: 382) and (b) the recently bolstered reputation and influence of outer borough New Yorker and North New Jersey politicians on the national stage (see Hall et al., 2016: 92).

Second, this study empirically examines claims about the uniqueness of Trump’s *in-your-face* rhetoric in U.S. politics by comparing him to a similarly styled regional figure who also ran for the presidency in 2016. Despite a few key differences between these two figures – Christie was a two-term moderate Republican New Jersey governor from 2010 to 2018 who was known for bipartisan cooperation (Racioppi, 2023), while Trump, a political outsider upon his entrance into the 2016 presidential race, is recognized by many as a populist figure who is comparable to Silvio Berlusconi of Italy and Viktor Orbán of Hungary (Moffitt, 2016; Muddle, 2022; Wodak et al., 2021) – explicit comparisons of Christie and Trump’s personas were common in political media in the early stages of the 2016 Republican primaries. One example of this is found in the summary of Christie’s 2016 campaign from NBC News in 2016:

Always telling it how it is, Christie was considered the most blunt candidate until Donald Trump joined the race. The governor’s inability to top Trump and granular support among conservatives and moderates led him to suspend his campaign in February.

(NBC News 2016)

Moreover, both of their performances in the Republican primaries received notable attention from both journalists (see Reston, 2015) and late-night comedy programs such as *Saturday Night Live*.^2^

To address these research questions, this study draws heavily on stylistics research (e.g., Culpeper, 2001; McIntyre and Bousfield, 2017; McIntyre and Culpeper, 2010) and impoliteness research (e.g., Bousfield, 2008; Culpeper, 2011; Tracy, 2017) because of the importance that impoliteness plays within this particular cultural schema (see Bousfield and McIntyre, 2011; Kozloff, 2000: Ch.6; Newman, 2014: Ch.5). Moreover, this approach to comparing the rhetoric of Christie and Trump from a stylistics standpoint also contributes essential insights into the long-observed trend that the boundary between politics and entertainment is becoming increasingly blurred. While it is certainly true that the discourse observed here involving Christie and Trump is not equal to fiction, I argue that televised political events are increasingly moving in the direction of entertainment due to external pressures on politics and journalism alike – for example, “infotainment” (Lakoff, 2005: 196) and “politicontainment” (Riegert, 2007).

This article has the following structure. The next section summarizes previous studies on impoliteness and characterization as well as the cultural schema of Metropolitan New Yorkers. The data section summarizes the journalist-made compilation videos that are used as data in the Christie and Trump case studies. The following two sections analyze these videos. The first one analyzes the data that precedes the 2016 Republican primary elections, and the second one analyzes the data from the 2016 Republican primary debates themselves. This two-part analysis provides an opportunity to see how these two figures transitioned from local figures to candidates for the U.S. presidency. It also opens up discussion about why, despite their similarities, Trump was able to secure the Republican nomination and eventually the presidency, while Christie only received modest gains in the primaries.

## 2. Impoliteness, characterization and the Metropolitan New Yorker tough-guy schema

### 2.1 Impoliteness and characterization in frontstage political activity types

This study adopts a ‘third wave’ approach to impoliteness (see Culpeper and Hardaker, 2017: 208), highlighting its tactical use by politicians to project strength and competence in entertaining ways. Thusly, it integrates classic and discursive approaches to (im)politeness (see Bousfield, 2010; Haugh and Culpeper, 2018). This approach conceptualizes impoliteness as strategic face-attacking behavior that generates conflict and disharmony (Bousfield, 2008); however, in certain contexts, it can also function to positively frame the speaker in the eyes of an overhearing audience (Tracy, 2017).

Impoliteness can be expressed in various ways. Both Bousfield (2008: 95) and Culpeper (2011) distinguish between on-record and off-record impoliteness, with Culpeper also identifying conventionalized formulae (Table 1) and implicational strategies (Table 2). These strategies will inform the analysis of the data that compares how Christie and Trump used impoliteness both prior to and during the 2016 Republican primary debates.

**Table 1.** Conventionalized impoliteness formulae. This is a simplified table taken from Culpeper (2011: 135–136).

Conventionalized impoliteness formulae	Example(s)
Insult (personalized negative vocatives)	*You fucking moron*
Insult (personalized negative assertions)	*You are such a bitch*
	*You can’t do anything right*
	*You disgust me*
Insult (personalized negative references)	*Your little arse*
Insult (personalized third person negative references in the hearing of the target	*The daft bimbo*
	*She’s nutzo*
Pointed criticism/complaints	*That is total crap*
Challenging or unpalatable questions and/or presuppositions	*Why do you make my life impossible?*
Condescensions	*That’s being babyish*
Message enforcers	*Listen here*
Dismissals	*Fuck off*
Silencers	*Shut the fuck up*
Threats	*I’m going to bust your fucking head off if you touch my car*
Curses and ill-wishes	*Fuck you*

**Table 2.** Implicational impoliteness categories. This is a simplified representation from Culpeper (2011: 155–156).

Implicational impoliteness strategy	Description
Form-driven	The surface form or semantic content of a behavior is marked (i.e., insinuation, innuendo, snide remarks, mimicry, etc.)
Convention-driven:	
(a) Internal	(a) The context projected by part of a behavior mismatches that projected by another part
(b) External	(b) External: The context projected by a behavior mismatches the context of use
Context driven	
(a) Unmarked behavior	(a) Unmarked behavior: An unmarked (with respect to surface form or semantic content) and conventionalized behavior mismatches the context (i.e., bald-on-record impoliteness)
(b) Absence of behavior	(b) Absence of behavior: The absence of a behavior mismatches the context

The notion of characterization is also essential to this study of the brash tough-guy schema of people (and politicians) from NYC and North New Jersey.^3^ While past studies on characterization have focused on fictional data, the argument taken here is that it can also be applied to ‘frontstage’ (see Goffman, 1959) political activities as well – as multiple studies have recently highlighted the fact that politicians are painstakingly prepped to project themselves in ways that are intended to impress targeted segments of the electorate (see Mullany, 2011; Tracy, 2017). Moreover, though it is a common trope for politicians from the Metropolitan New York area to be compared to fictional characters – particularly mobsters – in the press and in (late-night) comedies, the extant scholarly literature has largely overlooked how such representations reinforce cultural stereotypes to the point that real people, like politicians, take advantage of them for professional gains.^4^

Culpeper (2001: 35–36) argues that characterization is constructed through top-down and bottom-up cognitive processing and that this comprehension is cyclical – ‘what you see influences what you know, and what you know influences what you see’. Furthermore, Culpeper (2001: 75–83) notes three key schematic element categories for character types: personal traits, social roles and group memberships. He further emphasizes that a character’s interactions with others are critical for analysis because it shows how characters ‘position themselves relative to other characters, and how they manipulate others in pursuit of their goals’ (Culpeper, 2001: 251). Moreover, McIntyre and Bousfield (2017: 780) similarly assert that ‘the application of theories, models and frameworks for the analysis of (im)politeness can be revealing of processes of characterisation, and can also assist analysts in uncovering the locus of plot developments’.

However, these behaviors should also be analyzed in relation to the specific activity type(s) in which they occur. Levinson defines activity types as ‘a fuzzy category whose focal members are goal-defined, socially constituted, bounded events with *constraints* on participants, setting, and so on, but above all on the kinds of allowable contributions’ (1992 [1979]: 69, emphasis in original). McIntyre and Culpeper (2010) elaborate on this definition by stressing two essential dimensions of all activity types: the cognitive and interactional dimensions.

These insights have been successfully applied to the analysis of political communications already, particularly to the more institutionalized forms of frontstage political activities such as political press conferences (see Clayman and Loeb, 2018; Ekström and Eriksson, 2018: 345) and political debates (see Garcia-Pastor, 2008). Furthermore, frontstage political activity types have been identified as argumentative and combative by design because they revolve around politicians attempting to sell their agendas to potentially hostile audiences – be they fellow politicians, the press or members of the public. Moreover, Tracy (2017: 742) argues that ‘much political talk is designed for overhearing listeners even more than the actual party addressed,’ which therefore makes the use of impoliteness against certain collocutors useful in projecting a positive image of themselves (often via ‘reasonable hostility’). Additionally, impoliteness is particularly useful in political discourse because of its association with authenticity (Garcés-Conejos Blitvich, 2009: 287; Wodak et al., 2021: 387).

Issues surrounding adherence to the ‘allowable contributions’ (see also Thomas (2013 [1995]: 190–192) in frontstage political events are crucial for this analysis of Christie and Trump’s tactical use of impoliteness to gain political support. The extent to which Christie and Trump work within these formal constraints or push the boundaries of what is traditionally acceptable is discussed in detail in the case studies.

### 2.2 A few notes on the cultural schema of Metropolitan New Yorkers

The characterization of Metropolitan New Yorkers as direct and impolite has been perpetuated in popular culture for decades thanks in large part to popular fiction (e.g., *The Godfather* trilogy, *Goodfellas* and *The Sopranos*) and exploitative reality television programs (e.g., *Judge Judy*, *The Apprentice*, *Jersey Shore*, *The Real Housewives of New York/New Jersey* and *Mob Wives*). Central to all of these programs are the characters from NYC and North New Jersey who are brash, direct and impolite.

Newman (2014: 101–106) summarizes this distinctive New Yorker conversational style by analyzing a scene from the 1977 Woody Allen film *Annie Hall* that contrasts the interaction styles of a Jewish New Yorker, Alvy Singer, and his WASP girlfriend, Annie.^5^ In the famous split-screen dinner scene – juxtaposing Alvy’s Brooklyn family with Annie’s in Wisconsin – Newman emphasizes the differing priorities that New Yorkers and Midwesterners place on balancing their polite behavior/politeness in interaction, in particular the positive and negative politeness strategies described by Brown and Levinson (1987). He highlights how New Yorkers deemphasize negative politeness strategies while prioritizing positive politeness strategies – resulting in overlaps, fast pacing, expressiveness and engaging in personal topics (see also Tannen, 1981). Oppositely, Midwesterners lean toward negative politeness (e.g., waiting for pauses before speaking, indirectness in requests and avoidance of personal topics). He argues that the ‘high engagement’ style of New Yorkers often results in ‘arguments’ that are interpreted as banter or playfulness by locals (Culpeper et al., 2017; Haugh and Bousfield, 2012) but perceived as rude or hostile by outsiders – thereby underscoring broader cultural differences in conversational expectations between New Yorkers and American society at large (see ‘sociable arguments’ in Schiffrin, 1984). Similarly, Kozloff (2000: 206–207) observes that ‘talkativeness’ – characterized by short sentences, interruptions, repetition and taboo language – along with ‘non-WASPness’ (i.e., salient ties to specific immigrant communities) are defining features of the archetypal urban gangster. These characters are predominantly portrayed as coming from the peripheral regions of the New York Metropolitan area and speak with a nonstandard (Metropolitan) New York accent enriched by community-specific lexis. Trotta’s (2003) analysis of *The Sopranos*, reinforces the importance of talkativeness and non-WASPness in characterizing mafiosi from the Metropolitan New York area (Newark especially) by focusing on the Italian-American characters of this series who resist conventional ‘meddigan’ culture and construct identities as tough and street-smart criminals by employing a ‘low-status nonstandard language’ that is laced with vulgarisms and ‘colorful Italian-Americanisms’.^6^ The main character, Tony Soprano, is particularly celebrated for his witty, impolite style – as attested by countless entertainment journalists (see example below), fan websites and personal blogs.

Tony Soprano: There's an old Italian saying: you fuck up once, you lose two teeth.

(#22 of the 25 Best Tony Soprano lines from Ciriaco, 2025)

Investigating another work of fiction about New York gangsters, Bousfield and McIntyre (2011: 110) examine the famous ‘funny guy’ scene in the 1990 Scorsese film *Goodfellas* and show how the iconic character Tommy DeVito – as played by Joe Pesci – is characterized as having an aggressive and defiant persona that is built through the use of direct and unmitigated impoliteness. His repeated pointed questions and vulgar outbursts (see quote below) contribute to the construction of the quintessential New York mafioso character: stoic, defiant and highly aggressive.

Tommy DeVito: He says, ‘No, you're gonna tell me something today, tough guy. I said, ‘Alright, I'll tell you something, go fuck your mother.’ (Group laughter. Tommy makes punching motions towards his own head.) Bing! Pow! Boom! Bing!

(transcription conducted by Bousfield and McIntyre, 2011: 112–113)

Similar schematic elements have also been attributed to several prominent politicians from the region, though this is underexplored in the academic literature. For instance, former New York Mayor Ed Koch (1978–1989) was known for his sharp tongue – having become nationally recognized for calling people ‘whackos’, among other characteristically New York insults (O’Brien, 2016 [2005]: 58). Similarly, New York Governor Andrew Cuomo (2011–2021) rose to prominence through his combative style, especially so at the national level following his numerous disputes with President Trump (Breuninger, 2020). Chris Christie and Trump can be added to this list. Christie cultivated a brash, ‘Tough Guv’ persona (Christie, 2019: Ch.9) in public exchanges early in his tenure as governor and even proudly embraced comparisons to Tony Soprano (see Schwartz, 2019).^7^ Trump, a NYC businessman and cultural icon, was chosen to host *The Apprentice* precisely because of his colorful, tough and quintessential New York personality (O’Brien, 2016 [2005]: 14–15). In fact, this was even explicitly reinforced in the introduction sequence of the first episode, as given below:

New York. My city. Where the wheels of the global economy never stop turning. A concrete metropolis of unparalleled strength and purpose that drives the business world. Manhattan is a tough place. This island is the real jungle. If you’re not careful, it can chew you up and spit you out. But if you work hard, you can really hit it big. And I mean really big.

My name is Donald Trump, and I’m the largest real estate developer in New York. I own buildings all over the place […]

(as quoted in O’Brien, 2016 [2005]: 17)

The subsequent introduction sequences of seasons 1–11 additionally ended with the iconic Godfather quote, ‘It’s nothing personal. It’s just business,’ reinforcing his characterization as a tough guy with an intertextual reference to mobster fiction. Notably, the quote disappeared from the introduction in 2012 – the same year Trump began seriously considering a presidential run.^8^

As listed above, the most salient schematic elements (Culpeper, 2001: 75–83) that are central to the representation the Metropolitan New Yorkers listed above includes obvious group membership elements such as their regional places of origin (from the outer boroughs of NYC or North New Jersey) and their non-WASPness (i.e., immigrant family origins) – both of which are cued by the use of a non-standard Metropolitan New York accent. Additionally, key personal schematic elements include their talkativeness, brashness and a tendency towards impoliteness. However, applying these schematic elements to real-life figures from the Metropolitan New York region only gives a superficial picture of these figures. Moreover, it also does not explain why figures such as Christie and Trump would additionally rely on intertextual references to popular fictional characters from the region. Such a question is of central importance because this linking of their personas to mobster characters is not unique to Christie and Trump only. These associations have been made with other politicians such as Andrew Cuomo mentioned above and Former NYC Mayor and Trump lawyer Rudolf Giuliani (see Flegenheimer, 2021; Maher, 2018), as well as the general population of the region.^9^ For example, Alec MacGillis – a journalist for *The New Republic Magazine* – argued in an interview on CNN’s Erin Burnett OutFront that Tony Soprano ‘evokes New Jersey in a way that almost nothing else can’ because ‘Tony Soprano is one of the ultimate New Jersey icons’ (Burnett, 2014).

Naturally, this is not to say that *all* members of this community act in ways comparable to the schema discussed here. These descriptions of the Metropolitan New York conversational style should not be taken as absolute, but rather as an indication of a relative consensus of the community being considered (see Newman, 2014: 106). As Culpeper (2001: 79) explains, character/cultural schemas are shaped by both abstract social knowledge and specific personal experiences. None-the-less, it is also apparent from the summary above that the brash and straight-talking New Yorker/North Jerseyan schema is strong among both the local population and American society at large, particularly in pop culture. This in combination with the occupational pressures of contemporary politics (i.e., their social roles as politicians) to gain attention and popularity helps explain why some public figures from this region may choose to adopt such personas publicly.

## 3. Data: The compilation videos used in this study and their origins

The data used for this study comes from four compilation videos made by professional journalists. Compilation videos are useful for the purposes of investigating the tough-guy personas of Christie and Trump both prior to and during the 2016 primaries because (a) they contain a collection of excerpts of the most well-known instances of verbal sparring and digs involving both figures and (b) they were selected by journalists from mainstream publishers, the very people who were responsible for the dissemination of these iconic interactions in the first place. Moreover, as these video clips were compiled as a means of summarizing Christie and Trump’s most ionic conversational behaviors during key moments in their careers, it thus removes the role of the researcher in deciding which examples to analyze in detail. Naturally, using journalist-made compilation videos raises the potential issue that these excerpts represent exceptions of their communicative behaviors in these activity types rather than what is representative of them. However, given that the reputations of these two figures as tough guys from North New Jersey and NYC have been well established over many years by journalists, biographers, the general public and even Christie and Trump themselves, it is reasonable to consider these compilation videos a reliable sample of the kinds of behaviors for which they are well known – particularly in the frontstage political activities examined here. In fact, many of these very same video clips were not only beneficial to their professional progressions, but were even overtly promoted by themselves – including during the 2016 Republican primaries (see Alberta, 2019; Christie, 2019).

The first two videos showcase clips of highly publicized face-attacking exchanges by these figures before entering the 2016 Republican primaries. Each is about 1 minute in length. The video concerning Christie concentrates on many of his most notable interactions with the press, political colleagues and regular citizens that attracted significant media attention during his self-proclaimed ‘YouTube Governor’ era (i.e., his first term in office) during which his reputation as the ‘Tough Guv’ from New Jersey was solidified both locally and on the national stage (see Christie, 2019: Ch.9). This video from the YouTube channel of NJ.com is titled ‘Chris Christie maddest moments, from “sit down, shut up” to “idiot and numb nuts”’ (Donohue, 2015). The Trump video, titled ‘Donald Trump’s Most Disgusting Moments from The Apprentice,’ was compiled by Vulture/New York Magazine journalists and posted on the magazine’s website and YouTube channel (Halberstadt and Munro, 2016). This video compiles clips from notorious boardroom scenes from *The Apprentice* that received considerable entertainment press attention over the years. Credited for jolting his career after his numerous business setbacks of the 1990s and expanding his celebrity status, Trump’s television persona solidified his reputation as a straight-talking tough guy from NYC to a national audience (see D’Antonio, 2016: Ch. 13).^10^ Both of the videos in this section contain comments underneath. These comments will be briefly discussed in order to gauge the YouTube community’s reaction to them.

The next set of case studies examines two news videos that were compiled to summarize Christie and Trump’s 2016 primary debate performances. The video ‘Christie Interrupts’ (Mathis-Lilley, 2016) from Slate Magazine runs about 3.5 minutes. The video ‘Trump’s Memorable Republican Debate Moments’ (Frazier, 2023) from Politico is just under 2 minutes. Comment sections were not enabled for these videos.

## 4. Analysis of Chris Christie and Donald Trump prior to the 2016 primaries

### 4.1 ‘Chris Christie Maddest Moments’

The ‘Chris Christie Maddest Moments’ video contains eight clips of Christie sparring with his interlocutors from 2010 to 2014 while he was Governor of New Jersey. Half of them come from press conferences, while the others include a town hall meeting, a political rally, a televised Q&A forum and a public speech. As mentioned earlier, press conferences, town halls and other similar events that include Q&A segments are among the more institutionalized forms of political activity types.

I analyzed each clip and labeled all the variations of impoliteness behaviors that Christie used. As indicated in Tables 3 and 4, Christie utilizes a variety of conventionalized and implicational impoliteness strategies, often within a single turn/utterance (see 1 and 2 below).

**Table 3.** Conventional impoliteness in ‘Chris Christie maddest moments’ video.

Conventionalized formulae	#	Examples
Personalized insults	5	
• 2nd person insults	(3)	You must be the thinnest-skinned guy in America […] (Press Conference, May 2010)
• 3rd person insults	(2)	You have numb nuts like Reed G******* […] (Press Conference, Jan. 2012)
Unpalatable questions and/or presuppositions	2	Can you guys please take the bat out on her for once ? [*laughter*] (Press Conference, Apr. 2011)
Message enforcers	4	Let me tell you something! […] (Town Hall, Mar. 2012)
Dismissal	1	[…] first off, it’s none of your business. I don’t ask where you send your kids to school, so don’t bother me about mine (Call-in Q&A, Jun. 2011)
Silencer	1	[…] sit down and shut up! (Speech, Oct. 2014)
Threat	2	[…] after you graduate from law school, you conduct yourself like that in the court room, your rear-end’s gonna get thrown in jail […] (Town Hall, Jan. 2012)

**Table 4.** Implicational impoliteness in ‘Chris Christie maddest moments’ video.

Implicational impoliteness	#	Examples
Convention-driven: Internal	2	[…] something may go down tonight, but it ain’t gonna be jobs, sweetheart! [*audience cheers*] (Rally, Jan. 2012)
Form-driven: Mimicry & snide remark	2	Yet, she comes to you and is pining about, ‘*Oh my goodness! How awful this is!’* (Press conference, Apr. 2011)

Notably, personalized insults and message enforcers are the most used formulae. Concerning insults, Christie uses the following: ‘thinnest-skinned guy in America’ (see 1), ‘numb nuts’, ‘your rear end’, ‘idiot’ and ‘jerk’.

(1) Christie: You must be the thinnest-skinned guy in America [*laughter*]. Cause you think that’s a confrontational tone, then I – you know – you should really see me when I’m pissed! [*laughter*]

(Press Conference, January 2010)

When he uses implicational impoliteness in the clips, he used internal convention-driven strategies twice, as exemplified in (2). Another implicational strategy that he uses is form-driven. He does this via mimicry (see Table 4) and a snide remark, as seen in the second half of (1) above.

(2) Christie: So listen, you want to have the conversation later, I’m happy to have it, buddy. But until that time, sit down and shut up! [*audience cheers*]

(Public Speech, October 2014)

While the activity types that are represented in these video clips certainly include high potential for face-aggravating interactions between politicians and the press/concerned citizens, it is also noteworthy that the verbal attacks used by Christie against his targets differ from what would traditionally be expected from a governor participating in these formalized political activities. Therefore, Christie’s behaviors here are heavily foregrounded (see Leech, 1985). This is apparent by viewing the reactions of the audience in the clips and the alleged newsworthiness of these linguistic behaviors in the first place. In addition to this behavior being foregrounded, it is also evident that some people found Christie entertaining and even humorous (see discussion of incongruity humor theory in Simpson and Bousfield, 2017). Culpeper (2011: Ch.7) argues that impolite behaviors can serve a variety of functions, often simultaneously. This includes functions that (a) ostensively voice frustrations (affective impoliteness), (b) seek to gain or exercise power in a conversation over other participants (coercive impoliteness) or even (c) serve the additional function of entertaining other participants or overhearers (entertaining impoliteness).

Evidence that Christie’s behavior is considered humorously entertaining is found in the video footage itself and in the 108 comments below it. Concerning the YouTube commenters, nearly half of them indicated that they found him entertaining. Furthermore, I found 17 comments that made metadiscoursal comments that humorously pointed out how Christie’s utterances characterized him as a typical New Jerseyan. These range from explicit claims that he behaves like a typical New Jerseyan to humorous (intertextual) references (Fairclough, 2003: Ch. 3) to iconic films taking place in the Metropolitan New York area, including the Scorsese films *Goodfellas* and *Taxi Driver* (see comment quotes below).

• ‘He’s so New Jersey.’
• ‘Getthephuckouttahere!!!!’
• ‘He looks like someone straight out of Goodfellas.’
• ‘You talking to me? ….you you …..you talking to me?’

Concerning the audience members in the video clips themselves, there are multiple examples of them bursting into laughter when Christie was impolite. This is particularly the case in the clip where he called a reporter ‘the thinnest-skinned guy in America’. Such creative name-calling is more typical of informal activity types in which participants engage in jocular mockery (Haugh and Bousfield, 2012), thus making their usage all the more foregrounded in these formal political activities. Finally, Christie’s insults also set the stage for lifting up his own image as the fun and authentic participant and punching down his adversaries as overly serious and sensitive – thus simultaneously harnessing the coercive and entertaining impoliteness functions (see also superiority theory of humor in Billig, 2005: Ch.3). Painting a participant as overly serious while presenting oneself as able to *joke around* has been found to be an effective method of lifting one’s own public image and damaging the face of others (see Haugh, 2017).

Despite his mitigation strategies, his personalized attacks remained controversial and can be considered norm-challenging in the early 2010s (see Tracy, 2017: 744). This explains why these video clips became newsworthy in the first place. In fact, Christie’s aggression appears to follow the norms and expectations of more informal and jocular conversation, which explains why they were received with mixed reactions at the time. Furthermore, while Christie’s occasional combinations of aggression and humor in his outbursts are reminiscent of jocular mockery (Haugh and Bousfield, 2012) or even ‘banter’, it is essential to acknowledge that these cases analyzed here carry the primary function of boosting his own face needs rather than attending to social solidarity with his interlocutors (see discussion in Culpeper et al., 2017 on how banter-like behavior can be used to disguise the coercive impoliteness function).

### 4.2 ‘Donald Trump’s Most Disgusting Moments from The Apprentice’

The video ‘Donald Trump’s Most Disgusting Moments from The Apprentice’ contains six boardroom meeting scenes from *The Apprentice* from 2006 to 2015. The clips are taken from the original format of the show (Seasons 1–6, 10) and the *Celebrity Apprentice* format (Seasons 7–9, 11–15).

The boardroom meetings constitute a very distinguishable activity type. They occurred at the end of each episode and involved Trump and his advisors addressing the team that lost the competitive task earlier in the episode with the ultimate object of identifying and firing the weakest participant. The members of the losing team are thus put in a position where they must defend their own actions and deflect the blame for losing to someone else. Considering that Trump had no prior political experience before entering the 2016 Republican primaries, these boardroom interactions are the closest equivalent available for comparison with the Christie data. Moreover, analyzing these boardroom scenes provides a valuable basis for comparing the public personas of Christie and Trump prior the 2016 primaries because these segments played a key role in constructing Trump’s image as a successful, efficient and trustworthy executive – an image widely considered to have contributed to his perceived suitability for the presidency (Alberta, 2019: 213; Gabriel et al., 2018).

As exploitative reality television shows inherently utilize the humiliation of guests as a vehicle for entertainment (Culpeper, 2011: 249), face attack was commonplace in *The Apprentice* boardroom meetings by design. In this video compilation, Trump uses a variety of impoliteness strategies as indicated in Tables 5 and 6.

**Table 5.** Conventionalized impoliteness in ‘Donald Trump’s Most Disgusting Moments from The Apprentice’ video.

Conventionalized formulae	#	Examples
Pointed criticisms	1	How stupid is that, right? (Season 5, ep. 1)
Unpalatable questions and/or presuppositions	1	By interrupting me when I’m knocking him, what are you doing to yourself? (Season 5, ep. 1)
Dismissals	1	You’re fired! (All seasons)

**Table 6.** Implicational impoliteness in ‘Donald Trump’s Most Disgusting Moments from The Apprentice’ video.

Implicational impoliteness	#	Examples
Form-driven: Sexual innuendos/snide remarks	3	Must be a pretty picture, you falling to your knees. [*points to female contestant]* (Season 13, ep. 1)
Context-driven: Bald on record	1	He tried to outthink me and nobody outthinks me…nobody. (Season 14, ep.2)

Overall, Trump uses implicational impoliteness slightly more often in totality of the clips. Most of his implicational impoliteness strategies involve snide remarks or sexual innuendos involving the appearances of the contestants, particularly the female contestants (see Tables 6 and example 3 below). Another implicational example comes from an exchange where Trump teases his son about his hairstyle immediately before flaunting his own hair (see 4).

(3) Trump: By the way, much nicer now [*talking about a woman’s bottom*] that she lost all the weight.

(Season 11, ep. 8)

(4) Trump: Do you think that I should comb my hair like him [*points to his son*] […] You know it’s my hair! You know that! It’s a hair line. It’s actually a hairline.

(Season 13, ep. 2)^11^

Concerning conventionalized formulae, Trump delivers a pointed criticism, an unpalatable question and his now famous dismissal, ‘you’re fired!’ – which became Trump’s most recognizable catchphrase from the show. So far, the Trump data can be differentiated by the Christie data via his notable use of implicational impoliteness strategies. Another striking difference between the Trump and Christie data is that this data shows him targeting the physical appearances (and sexuality) of his interlocutors, which is a particularly central and sensitive aspect of a person’s ‘quality face’ (see Spencer-Oatey, 2008). Another notable difference is that Trump also discusses his own appearance and explicitly brags about himself.

Despite his strong links with the NYC tough-guy image in the early 2000s, the commenters of this video do not seem to explicitly associate Trump with his home city or this character trope, though it is notable that most of the commenters found Trump entertaining. Scanning through the 326 YouTube comments, I did not identify a single comment that characterized his behavior being typical of a New Yorker or even mention NYC at all. This finding also differentiates the Trump and Christie data. While Christie’s behavior is regularly and explicitly associated with New Jersey and the tough guy image by newscasters and online commenters alike, Trump appears to have lost some of the ostensive association with it by the time this video was made in 2016. This is perhaps due to Trump’s now decades-long exposure to the American public via his flashy businessman and image as a reality television star.

### 4.3 Discussion of the ‘Chris Christie Maddest Moments’ and ‘Trump’s Most Disgusting Moments from The Apprentice’ videos

The analyses of these videos indicate how Christie and Trump took advantage of strategic impoliteness strategies in these highly publicized exchanges in ways that are reminiscent of the talkative and brash stereotypes of people from NYC and North New Jersey. For Christie, this connection with North New Jersey was salient, even at the time when the video was released by NJ.com in June 2015. Indeed, much of the impolite language used by Christie contained sportily (and informal-like) elements that were heavily foregrounded and entertained many onlookers to the point that his outbursts became newsworthy. This strategy is also evident in his choice of conventionalized formulae. Even though these clips showed Christie using them much more often than Trump did, the formulae that he does use – for example, the personalized insults ‘thinnest-skinned guy in America’, ‘numb nuts’ and ‘idiot’ – are fairly mild in terms of impoliteness gravity (cf. Jay, 1992: Ch.5). These uses of mild insults thus provide an indication that Christie is accommodating his behavior to the norms against taboo language in publicized political activities somewhat, while still maintaining the image of a quintessentially brash, confrontational yet witty New Jerseyan – much in the image of Tony Soprano to whom Christie is so often compared. This appears to have worked, as comparisons to fictional mobsters persisted in both his media coverage and in YouTube comments of the video.

Trump’s brash and direct NYC persona was a key factor in his selection as host of *The Apprentice* in the early 2000s. By 2016, however, the New Yorker element seemed less ostensive. Still, his conversational style in the sampled video clips retains many of the tough-guy New Yorker schematic elements. What is unique about Trump compared to Christie, however, is his use of implicational impoliteness. As Schubert (2025: 16) notes, mob bosses in Scorsese films often use indirectness to exert power, maintain secrecy and allow plausible deniability. Trump appears to mirror these strategies, particularly in his remarks that targeted the appearances (including sexual innuendos) of female contestants. His unmitigated use of self-aggrandizing comments is also reminiscent of fictional tough-guy characters (see Kozloff, 2000: 213). Such behaviors – attacking appearances, violating modesty norms and excessive bragging – are socially tabooed and strongly negatively marked for the general American population (see Allan and Burridge, 2006; Culpeper et al., 2017). However, Trump appears to make it work in his favor – likely because of his consciously constructed image of a NYC real-estate mogul and tough-boss in *The Apprentice*.

## 5. Analysis of Chris Christie and Donald Trump in the Republican primary debates

Before analyzing Christie and Trump’s performances in the debates, it is first necessary to explain the format of this activity type. American political debates have a rigid format that are explicitly explained before each one commences. Though some details varied between individual debates (e.g., such as the amount of time allocated for answers and follow-ups), I have identified the following elements in all of the 12 official 2016 Republican primary debates.^12^

• **Setting:** Convention hall or university auditorium with a live audience.
• **Participants:** Candidates and moderators.
• **Rules:** Moderators choose topics and speakers; response times are fixed and enforced.
• **(Im)politeness Norms (i.e., allowable contributions):** Candidates should defend their views and contrast their records respectfully; personal attacks are considered out of bounds. Moderators intervene as needed.
• **Register:** Formal language and business attire is the norm.

The following two subsections examine the data that summarize Christie and Trump’s performances in these debates. The discussion subsection contextualizes these findings.

### 5.1 ‘Christie Interrupts’

The ‘Christie Interrupts’ video contains five clips from five of the Republican primary debates. As shown in Tables 7 and 8, Christie uses impoliteness differently in this data set compared to what was seen in the New Jersey data.

**Table 7.** Conventionalized impoliteness from ‘Christie interrupts’ video.

Conventionalized formulae	#	Examples
Personalized insults: 2nd person insult	2	No, you already had your chance, Marco, and you blew it. (6th Debate, Jan 2016)
Unpalatable questions	3	We have — […] and we’re talking about fantasy football? Can we stop? (3rd Debate, Oct. 2015)
Message enforcers	2	Carly, listen […] (2nd Debate, Sep. 2015)
Condescensions	2	Let’s start talking about those issues tonight and stop this childish back-and-forth between the two of you! (2nd Debate, Sep. 2015)

**Table 8.** Implicational impoliteness from ‘Christie interrupts’ video.

Implicational impoliteness	#	Examples
Convention-driven: External (Sarcasm)	2	Maria, I’d like to interrupt this debate on the floor of the Senate to actually answer the question you asked[…] (6th Debate, Jan 2016
Context-driven: Bald on record	2	Nobody in America cares about that. (5th Debate, Dec. 2015)

In this data set, Christie only uses two personalized insults (i.e., ‘you’re […] just blowing hot air’ and ‘you blew it’) that are noticeably even milder than the insults that he uses in the ‘Chris Christie Maddest Moments’ video. Christie also uses unpalatable questions, condescensions and message enforcers (see Table 7). Regarding the implicational strategies shown in Table 8, he uses sarcasm and context-driven strategies twice each. Overall, his face attacks emphasize that his work experience is more directly relevant to the presidency than that of his many senatorial rivals.

The data suggests that Christie has adjusted how he uses impoliteness to the context and allowable contributions of nationally televised political debates. He concentrates his face attack on the rhetoric used by his targets and/or their past professional experiences. Moreover, this data shows less reliance on personalized insults against individuals. This was likely an intentional strategy on his part to continue portraying himself as a New Jersey tough-guy politician – an image that helped him gain national notoriety in the first place – but in a way that was toned down for a broader American audience. Christie’s awareness to differing cultural views on (im)politeness is evident in the third debate (Oct. 28, 2015) – during an exchange that is not included in the compilation – in which he highlights these differences while complaining to a moderator about his repeated interruptions:

Christie: No, John. John, do you want me to answer, or do you want to answer? [*laughter*] How are we going to do this? [*applause*] Because I’ve got to tell you the truth, even in New Jersey what you’re doing is called rude! […] [*laughter*]

(American Presidency Project, 2015)

Christie’s use of the conventional implicature *even in X* (Grice, 1969), in reference to the (im)politeness norms of his home state, provides evidence that he is not only aware of the particularities of the New Jersey conversational style in comparison to the rest of the country, but also presupposes that the general American public is aware of it too. Through this self-deprecating comment, Christie continues to approach conflict in his frontstage activities playfully and with humor, as was also seen in the ‘Chris Christie Maddest Moments’ video. This is apparent from the frequent laughter that follows his turns and the reactions of some of the people targeted by his face-aggravating utterances, who occasionally laughed off his attacks.^13^ Overall, the targets of Christie face-aggravating utterances appeared to have received them more lightheartedly compared to their reactions to Trump’s verbal attacks, as shown in the analyis of Trump's debate performances below.

### 5.2 ‘Trump’s Memorable Republican Debate Moments’

‘Trump’s Memorable Republican Debate Moments’ contains seven clips from five of the Republican debates. Trump’s face attacks in this data mirror patterns seen in *The Apprentice* data, and his attacks against his opponents are frequently *ad hominem* in nature (see Mercieca, 2020: Ch.4).

Tables 9 and 10 show that Trump continues to rely on a variety of implicational strategies, though his uses of conventionalized formulae are more pronounced here than in *The Apprentice* data. For instance, there are two examples of Trump using creative personalized insult formulae, which intriguingly will later develop into one of Trump’s most emblematic rhetorical strategies as a politician (see Tyrkkö and Frisk, 2020). One such insult, ‘Little Marco’, targets Senator Rubio’s height and perceived lack of political stature. He also negatively references the appearance, gender and actions of Megyn Kelly, one of the moderators of the first debate (See Table 9).

**Table 9.** Conventionalized impoliteness from ‘Trump’s Memorable Republican Debate Moments’ video.

Conventionalized formulae	#	Examples
Personalized insults	2	
• 2nd person insult (vocative)	(1)	Don’t worry about it, little Marco! (11th Debate, March 2016)
• 3rd person insult (negative reference)	(1)	There was blood coming out of her eyes, blood coming out of her …wherever. (Post 1st Debate, Aug. 2015)
Pointed criticism/complaints	3	[…] it was such a disaster, those last 3 months, that Abraham Lincoln couldn’t have been elected! (9th Debate, Feb. 2016).
Message enforcer	3	[…] remember that! (9th Debate, Feb. 2016)
Silencer	1	A lot of times — let me talk. [*makes a Shh motion*] Quiet! [*audience boos*] (8^th^ debate, Feb. 2016)

**Table 10.** Implicational impoliteness from ‘Trump’s Memorable Republican Debate Moments’ video.

Implicational impoliteness	#	Examples
Convention-driven	4	
Internal	(3)	I never attacked him on his look, and believe me, there’s plenty of subject matter right there. [*laughter*] That I can tell you. (2^nd^ debate, Sep. 2015)
External (Sarcasm)	(1)	Moderator: You’ve called women you don’t like fat pigs [*laughter*] […]
		Trump: Only Rosie O’Donnell! [*laughter and applause*] (1st Debate, Aug. 2015)
Form-driven: Snide remark	1	[…]And he referred to my hands, if they are small, something else must be small. I guarantee you there is no problem. I guarantee. [*laughter*] (11th Debate, Mar. 2016)
Context-driven: Bald on record	1	The world Trade center came down during your brother’s reign […] [*audience boos*] (9th Debate, Feb. 2016)

Trump also uses implicature to belittle a contestant's appearance via *paralipsis* (see Mercieca, 2020: Ch. 9) by denying he mocked Senator Paul’s ‘look’ while implying simultaneously that there is indeed something about it worth mocking (see Table 10). Trump also indicates a willingness to attack the close relations of his opponents. In two examples, Trump verbally attacked Jeb Bush via his older brother, former President George W. Bush (see Table 10 and example 5 below).

(5) Trump: Your brother's administration gave us Barack Obama because it was such a disaster, those last three months, that Abraham Lincoln couldn't have been elected! [*laughter*]

(9^th^ Debate, Feb. 2016)

Overall, Trump does not appear to have made accommodations to align his preferred impoliteness strategies with the traditional allowable contributions of formal political debates. This is especially apparent in his methods of attacking the more sensitive aspects of face such as the appearances and family relations of his opponents (see Culpeper, 2011: 25). Evidence that Trump’s impoliteness is evaluated as more offensive than Christie’s in the debate data can be found in the facial expressions of his interlocutors, vocal complaints/reciprocations and the occasional booing coming from the audience members. However, Trump’s impolite utterances also received laughter and applause as well. In fact, sometimes various segments of the audience reacted differently to the same behaviors and the booing and laughter/applause overlapped.

### 5.3 Discussion of Republican primary debate case studies

The analyses of the ‘Christie Interrupts’ and ‘Trump's Memorable Republican Debate Moments’ videos show that Christie adjusted his behaviors somewhat when participating in the primary debates, while Trump’s strategies were similar across the two data sets. In his 2019 memoir, Christie reflects on the similarities and differences between them during the primaries, stating:

From a stylistic perspective, he was everything I was – but on jet fuel. He was brash. He was direct. He was in your face. I was doing all the things I normally did and was good at doing. I was getting strong reviews from the pundits. Yet he was still dominating.

(Christie, 2019: para. 394)

The differing levels to which Christie and Trump ostensively referenced their New York/North New Jersey tough-guy personas during the primaries also give some hints of the strengths and weaknesses of projecting them in national politics. The analyses above show how this persona is effective in projecting strength, genuineness and even a sense of humor. However, despite the recent improvements in the perceptions of Metropolitan New Yorkers (see Hall et al., 2016: 92), many segments of American society still evaluate New Yorkers/New Jerseyans negatively because of their association with aggressiveness and *otherness* compared to the general American society (Labov, 2006: Ch. 13). Trump appeared to overcome these challenges with the electorate thanks to his personalized brand of celebrity that never-the-less continues to utilize many of the key features of the NYC tough-guy persona. Particularly, Trump appears to adopt a discourse style that has notable similarities with hypermasculinized fictional mob bosses in that it targets highly sensitive aspects of the faces of his interlocutors via a variety of impoliteness strategies, including a variety of implicational ones (see Schubert, 2025).^14^ On the other hand, Christie’s ostensive association with New Jersey as its governor and his characterization as a stereotypical New Jerseyan may have helped him capture national attention and even amuse many spectators of the debates, but it is also apparent that it did not help him capture votes in 2016. It is notable that he never gained more than 10% of the vote in any individual primary.

As an interesting sidenote, both Christie and Trump ran in the 2024 Republican primaries, with results that largely mirrored those of 2016. Both maintained personas similar to those they projected in 2016, though Christie notably adopted an explicitly anti-Trump stance. Lacking sufficient support, Christie dropped out just days before the Iowa caucus. Trump ultimately secured both the Republican nomination and the presidency.

## 6. Final discussion and conclusions

This study shows how politicians can strategically draw on cultural schema to craft personas in an attempt to capture attention and foster positive self-presentation. Both Christie and Trump exemplify this by characterizing themselves as New York/North New Jersey tough guys in publicized events, though in distinct ways. Christie adapted his tough-guy persona from his gubernatorial years to the 2016 presidential race, probably in an attempt to tailor it to be more suitable for a national audience, while consistently emphasizing his *New Jerseyness*. Trump, by contrast, maintained a more consistent brash persona from reality TV to politics, marked by more severe face attacks and creative use of implicational impoliteness. Unlike Christie, Trump backgrounded explicit references to NYC during his campaign, though he retained a recognizable NYC discourse style that was talkative, brash and impolite. These differences underscore the uniquely confrontational and norm-challenging nature of Trump’s 2016 performances, even when compared to Christie.

From a stylistics standpoint, one of the most intriguing connections between these two politicians was that they were both likened to fictional mobster characters. Interestingly, these comparisons and references to mobster characters continued after 2016 as can be found in the media, late-night television and social media. Late-night comedian Bill Maher even ran an entire segment titled ‘Married to the Mob’ in 2018 that humorously argued that Trump’s administration was run like a New York mob family – making numerous references to Scorsese films, *The Godfather* and *The Sopranos*. Among other things, he called Trump ‘Childish Gambino’ and Chris Christie ‘the fat guy from New Jersey who he (i.e., Trump) keeps around to make himself look thin’, an obvious reference to Tony Soprano.

Future research could expand on these findings by examining other politicians from the Metropolitan New York area. Andrew Cuomo’s unexpected and unsuccessful bid in the 2025 NYC mayoral race – after resigning as governor in 2021 amid multiple sexual misconduct allegations – offers a potential avenue for further research (see Pretsky et al., 2025). Moreover, similar investigations of politicians from other communities that are well represented in popular media (e.g., Californians, Chicagoans, Bostonians, Texans, etc.) would also be of vital importance in this area of pragmastylistic inquiry. California Governor Gavin Newsom, who in recent years has adopted a confrontational style often likened to Trump’s to challenge him and other MAGA Republican politicians, provides one potential avenue in this direction (see Sullivan, 2025).

## Notes

1. Though a life-long New Yorker, Trump has since moved his permanent residency to Florida in 2019. However, he still maintains his properties in New York.

2. One such parody of the primary debates can be found on Saturday Night Live’s YouTube Channel here: https://youtu.be/r0Th70moeuc?si=tDRO4jfh5QjWw8oI.

3. A gender-neutral alternative to *tough guy* is *tough cookie*. However, the masculine term is maintained because Trump and Christie have ostensively been described as *tough guys*.

4. However, real-life Mafiosi have been observed imitating fictional portrayals of mob behavior (Renga, 2011: 8).

5. WASP is an acronym for *White Anglo-Saxon Protestant*. They represent the most privileged class of people in the U.S.

6. Italian Americans use *meddigan* to denote mainstream white (non-Italian) Americans.

7. Christie himself positively embraces a comparison with Tony Soprano in a Piers Morgan Tonight (CNN) interview from June 14, 2011, as documented in the official CNN transcript: https://transcripts.cnn.com/show/pmt/date/2011-06-14/segment/01.

8. This was verified by watching all introduction sequences for each season of *The Apprentice*.

9. Trump is unique on this list of politicians because he is the only one without Italian-American ancestry. Like the others, however, he comes from an immigrant family that settled in one of the outer boroughs of NYC (Queens). He is also unique in having longstanding allegations of ties to New York mafia figures (see Barrett, 2016 [1992]).

10. Trump also projected this same persona as Vince McMahon’s on-screen rival in WWE (World Wrestling Entertainment) from 2007 to 2009, most notably in the *Battle of the Billionaires* at WrestleMania 23 in 2007. For details, see Trump’s WWE biography on their webpage: https://www.wwe.com/superstars/donald-trump.

11. I treat this as mild impoliteness, as Trump punches down on his son’s hairstyle to favorably contrast his own (cf. Bousfield, 2010 on ‘rudeness’), though it is certainly debatable whether this tease serves a primarily prosocial or antisocial function (see Haugh, 2017).

12. Garcia-Pastor (2008: 121) argues that traditional debates have a zero-sum essence that encourages debaters to exercise power by using impoliteness to discredit their opponents and coerce them into specific unflattering courses of action.

13. See Bousfield and McIntyre (2011) for a discussion on the need to adapt pragmastylistic methods to analyze (im)politeness across verbal, paralinguistic and non-verbal cues in multimodal texts.

14. However, Lakoff (2017: 598) underscores an irony of Trump’s ‘macho’ persona by highlighting the effeminate qualities of his gestures (see also Hart, 2024), his use of hyperbole and his gossip-like speech. Kozloff (2000: 213) identifies similar tensions in the portrayals of mobsters in film, who are depicted as hypermasculine yet simultaneously characterized by stereotypically feminine traits such as vanity, flamboyance and emotional expressiveness. Trump’s affinity for the Village People has likewise attracted ironic critiques (Rees, 2025).
